# Lipid metabolism-related gene signature predicts prognosis and unveils novel anti-tumor drugs in specific type of diffuse large B cell lymphoma

**DOI:** 10.1186/s10020-024-00988-4

**Published:** 2024-11-13

**Authors:** Cancan Wang, Ran Zhang, Huan Zhang, Haixia Gao, Yubing Zhu, Lichao Jiao, Zhiqiang Yi, Meiyu Zhou, Xinxia Li

**Affiliations:** 1 Department of Pathology, Xinjiang Medical University Affiliated Tumor Hospital, Urumqi, China; 2 Chongqing University Fuling Hospital, Chongqing, China

**Keywords:** Lipid metabolism, DLBCL, Tumor immune microenvironment, Prognosis

## Abstract

**Background:**

Diffuse large B-cell lymphoma (DLBCL) is the most common type of lymphoma which possess highly aggressive and heterogeneous. Despite advances in understanding heterogeneity and development of novel targeted agents, the prognosis of DLBCL patients remains unsatisfied. Lipids are crucial components of biological membranes and signal transduction while accumulating evidence has supported the vital roles of abnormal lipid metabolism in tumorigenesis. Furthermore, some related pathways could serve as prognostic biomarkers and potential therapeutic targets. However, the clinical significance of abnormal lipid metabolism reprogramming in DLBCL has not been investigated. In the current study, we developed a prognostic risk model for DLBCL based on the abnormal expressed lipid metabolism genes and moreover based on our risk model we classified patients with DLBCL into novel subtypes and identified potential drugs for DLBCL patients with certain lipid metabolism profiles.

**Methods:**

We utilized univariate Cox regression analysis to identify the prognosis-related lipid metabolism genes, and then performed LASSO Cox regression to identify prognostic related lipid metabolism related genes. Multivariate cox regression was used to establish the prognostic model. Patients were divided in to high and low risk groups based on the median risk score. Immune cell infiltration and GSEA were used to identify the pathways between high and low risk groups. Oncopredict algorithm was utilized to identify potential drug for high-risk patients. In vitro cell apoptosis and viability analysis were employed to verify the specific tumor inhibition effects of AZD5153.

**Results:**

Nineteen survival related lipid metabolism genes TMEM176B, LAYN, RAB6B, MMP9, ATAD3B, SLC2A11, CD3E, SLIT2, SLC2A13, SLC43A3, CD6, SIRPG, NEK6, LCP2, CTTN, CXCL2, SNX22, BCL6 and FABP4 were identified and subjected to build the prognostic model which was further verified in *four* external microarray cohorts and one RNA seq cohorts. Tumor immune microenvironment analysis and GSEA results showed that the activation of MYC targets genes rather than immunosuppression contribute to the poor survival outcome of patients in the high-risk group. AZD5153, a novel bivalent BET bromodomain inhibitor which could inhibit the transcription of MYC and E2F exhibited specific antitumor function for cells with high-risk score.

**Conclusions:**

Our results provide the first lipid metabolism-based gene signature for predicting the survival of patients with DLBCL. Furthermore, by determining novel subtypes with our lipid metabolism prognostic model we illustrated that drugs that compromising MYC target genes rather than immune checkpoint inhibitors may be beneficial to DLBCL patients with certain lipid metabolism profiles.

**Supplementary Information:**

The online version contains supplementary material available at 10.1186/s10020-024-00988-4.

## Introduction

The most common and aggressive form of B cell lymphoma Diffuse large B-cell lymphoma (DLBCL) is featured by highly heterogeneous (Sehn and Salles 2021). The widely used “cell-of-origin” (COO) methodology defines two prominent subtypes termed germinal center B cell-like (GCB) and activated B cell-like (ABC) (Poletto et al. 2022). Nonetheless, the COO distinction does not fully account for the heterogeneous clinical outcomes and drug responses (Poletto et al. 2022; Wright et al. 2020). Currently, although the R-CHOP (rituximab in combination with CHOP) chemotherapy is widely used as the first line regimen, approximately 30–40% relapse within the first 2 years of diagnosis (Coiffier et al. 2010; Tavakkoli and Barta 2023). Furthermore, first-line multiagent immunochemotherapy also fails to elicit a durable response in approximately one-third of patients with DLBCL (Schmitt et al. 2023). In this regard, the highly heterogeneous in terms of molecular and histological features makes it difficult to predict prognosis and decide therapeutic strategies for patients with DLBCL (Nastoupil and Bartlett 2023; Hilton et al. 2023) thus posing major challenges in DLBCL treatment. Therefore, a comprehensive understanding of its molecular makeup is an urgent need to accurately predict the prognosis and develop specific therapeutic drugs.

Metabolic reprogramming represents one of the hall marks of cancers by facilitating tumor proliferation, invasion, and metastasis (Xiao et al. 2023; Hanahan 2022). Among the three major metabolism pathways, lipid metabolism alteration is the most prominent metabolic alterations in cancer development (Jin et al. 2023; Gong et al. 2022; Yang et al. 2023; Broadfield et al. 2021). Lipids, including fatty acids, triglycerides, phospholipids et al. are not only vital components of biological membranes and energy resource but also play critical roles in cellular signaling pathway and shaping the tumor microenvironment (Lim et al. 2022; Grabner et al. 2021). For example, tumor cells upregulate lipogenesis, and fatty acid oxidation (FAO) to produce energy for proliferation (Koundouros and Poulogiannis 2020; Xu et al. 2021). Cancer stem cells maintain their stemness by reprogramming lipid metabolism(Yi et al. 2018). Lipids could also regulate the fate of T cells at the transcriptional, epigenetic, and post-translational levels (Lim et al. 2022). Therefore, abnormal lipid metabolism could be prognostic biomarkers and targeting the lipid metabolism regulating pathway has been regarded as a promising strategy in cancer treatment (Bian et al. 2021).

Nevertheless, the possible clinical significance of abnormal lipid metabolism in DLBCL remains undetermined. Therefore, to better understand the association between altered lipid metabolism and clinical outcome of DLBCL, we comprehensively investigated the expression pattern of lipid metabolism genes in DLBCL by integrating survival data and gene expression from GEO and TCGA datasets. Based on lipid metabolism-based gene signature, we provide a model with high predictive efficacy for predicting the survival of patients with DLBCL. Furthermore, using the lipid metabolism prognostic model we determined novel subtypes featured by distinct lipid metabolism profiles and found that drugs that compromising MYC target genes rather than immune checkpoint inhibitors may be beneficial to DLBCL patients with specific lipid metabolism status.

## Materials and methods

### Data acquisition

Microarray and RNA-seq gene expression and the relevant prognostic and clinicopathological data of DLBCL were downloaded from the public database Gene Expression Omnibus (GEO) (https://www.ncbi.nlm.nih.gov/geo/.) (GSE10846, GSE31312, GSE181063, GSE32918, *GSE53786* and GSE56315) and UCSC Xena (https://xenabrowser.net/datapages/) (TCGA_DLBCL). GSE56315 data set which contains 55 tumor samples and 33 normal samples was used to identify differentially expressed genes (DEGs). After including samples with the criteria (1) histologically confirmed DLBCL; (2) initially treated with RCHOP or CHOP regimen; (3) overall survival (OS) time more than one month. The GSE181063, GSE10846, GSE31312, GSE32918 and *GSE53786* included 811(Table S1), 400 (Table S2), 466(Table S3), 154 (Table S4) and 113 (Table S5) tumor samples respectively.

### Lipid metabolism-related genes (LMRGs) preparation

A total of 7286 LMRGs (Table S6) were obtained from the Molecular Signature Database (MsigDB https://www.gsea-msigdb.org/) (22) by interesting 176 individual collections (Table S7).

### Identification of DEGs between DLBCL and normal tissue

The “Limma” package was used to identify GEGs in 55 DLBCL samples and 33 normal samples. *P* < 0.01 and |log_2_ fold change (FC)|> 2 were utilized as the selection criteria.

### Screening of prognostic related LMRGs

We first interested DEGs and LMRGs and then applied univariate Cox regression to identify prognostic related LMRGs using the GSE181063 data set (*N* = 811) with the criteria of *P* < 0.05 and 238 LMRGs were obtained. Then least absolute shrinkage and selection operator (LASSO) Cox regression model with 10-fold cross validation was further utilized to select the most powerful prognostic genes by minimizing the risk of over-fitting.

### Prognostic model construction and validation

19 LMRGs were left to build a prognostic model using multivariate Cox regression and this model was further valid using 4 external microarray data sets GSE10846 (*N* = 400), GSE31312 (*N* = 466), GSE32918 (*N* = 154) and GSE53786 (*N* = 113) and RNA seq data set TCGA_DLBCL (*N* = 46).

### Tumor microenvironment and immune cell infiltration analysis

The single-sample gene set enrichment analysis (ssGSEA) was performed to quantify the proportions and distributions of tumor-infiltrating immune cells (TIICs) with “GSVA” package in R. We also calculated the immune, stromal and Estimate scores with “ESTIMATE” package in R. The expression of immune checkpoint molecules (PDL1,CTLA4,HAVCR2) were evaluated between high and low risk groups.

### Functional analysis of DEGs between high and low risk groups

GESA analysis using the gene list (h.all.v2023.2.Hs.symbols) was applied to investigate the alteration pathways between high and low risk groups.

### Drug sensitivity analysis

Using the oncopredict package in R software, the sensitivity score of each small molecule compound was calculated for each patient in the high-risk group and low-risk group. Then, we used PubChem (https://pubchem.ncbi.nlm.nih.gov/) website to visualize the conformations of drugs in 3D.

### Risk analysis of DLBCL cell lines

The expression data of seventeen DLBCL cell lines were downloaded from Cancer Cell Line Encyclopedia (CCLE, https://portals.broadinstitute.org/ccle/data). Cells were divided into high and low risk groups based on the LMRGs survival model.

### Cell viability analysis

Human B cell lymphoma cells DOHH2 and Su-DHL-6 were purchased from the Cell Bank of the Chinese Academy of Science (Shanghai, China) and were cultivated in RPMI- 1640 (Hyclone) supplemented containing 2 mM L-glutamine and 10% FBS (Life Technologies). DOHH2 (High risk) and SU-DHL-6 (Low risk) cells were seeded into 96-well plates and subjected to AZD5153 treatment (10 nM,50 nM,100 nM). The cell viability was acquired at indicated time points using the CCK8 kit (BA00208, Bioss, China).

### Cell apoptosis analysis

Apoptosis was determined by using FITC Annexin V Apoptosis Detection Kit (556547, BD Pharmingen, USA). Briefly, DOHH2 and SU-DHL-6 cells treated with 200 nM AZD5153 were diluted at a concentration of 10^6^ cells/ml and stained with Annexin V and PI and subjected to flowcytometry analysis (DxFLEX, Beckmancoulter USA). The relative portion of Annexin V-positive cells was determined using the Flowjo software.

### Statistical analysis

Statistical analysis was carried out in R version 4.4.0. Log-rank test was used for univariate Cox regression analysis. Wilcoxon test was performed to assess the statistical significance between two groups for the bioinformatic analysis and Student t test was applied for the cell apoptosis analysis. *P* < 0.05 denoted as statistically significant.

## Results

### Determination of differentially expressed LMRGs between normal and DLBCL samples

**Fig. 1:**
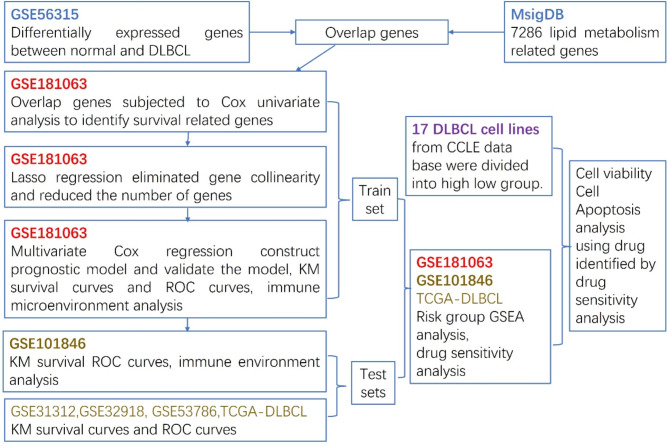
The flowchart of our research process

The flow chart shows the overall experimental design of this study (Fig. 1). A total of 7286 LMRGs (Table S6) were obtained from the Molecular Signature Database (MsigDB https://www.gsea-msigdb.org/) (22) by interesting 176 individual collections (Table S7). We then screened the DEGs between normal and DLBCL patients in GSE56315 and a total of 2972 DEGs were identified and intersected with 7286 LMRGs and finally obtained 1142 differentially expressed LMRGs.

### Development and validation of the prognostic related LMRGs model

**Fig. 2:**
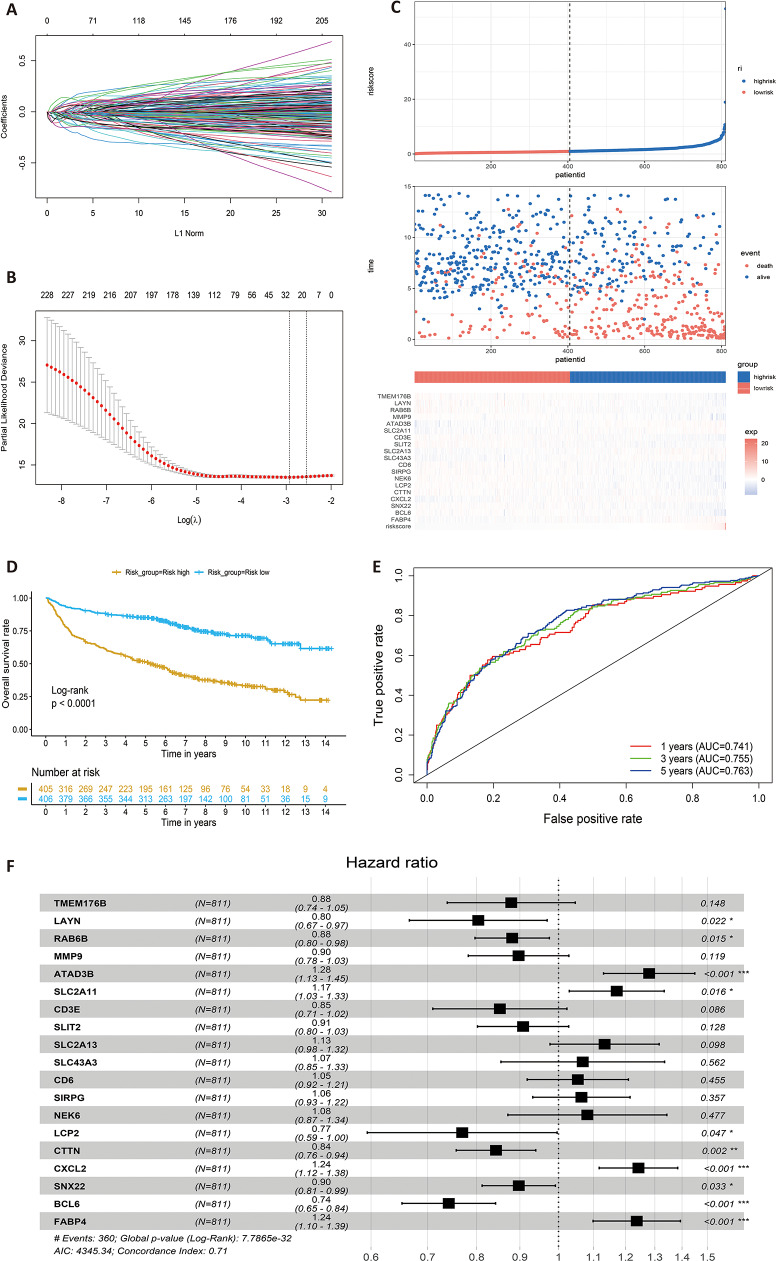
Identification of lipid metabolism related prognostic genes in DLBCL patients. (**A**,**B**) 19 Lipid metabolism related candidate genes were selected by LASSO Cox regression. (**C**) 811 patients in GSE181063 were divided into high and low risk group according to the median of risk score. (**D**) Kaplan-Meier analysis of overall survival in high and low risk groups. (**E**) Time-dependent ROC analysis of the lipid metabolism risk model. (**F**) Forest plot of 19 lipid metabolism related genes

**Fig. 3:**
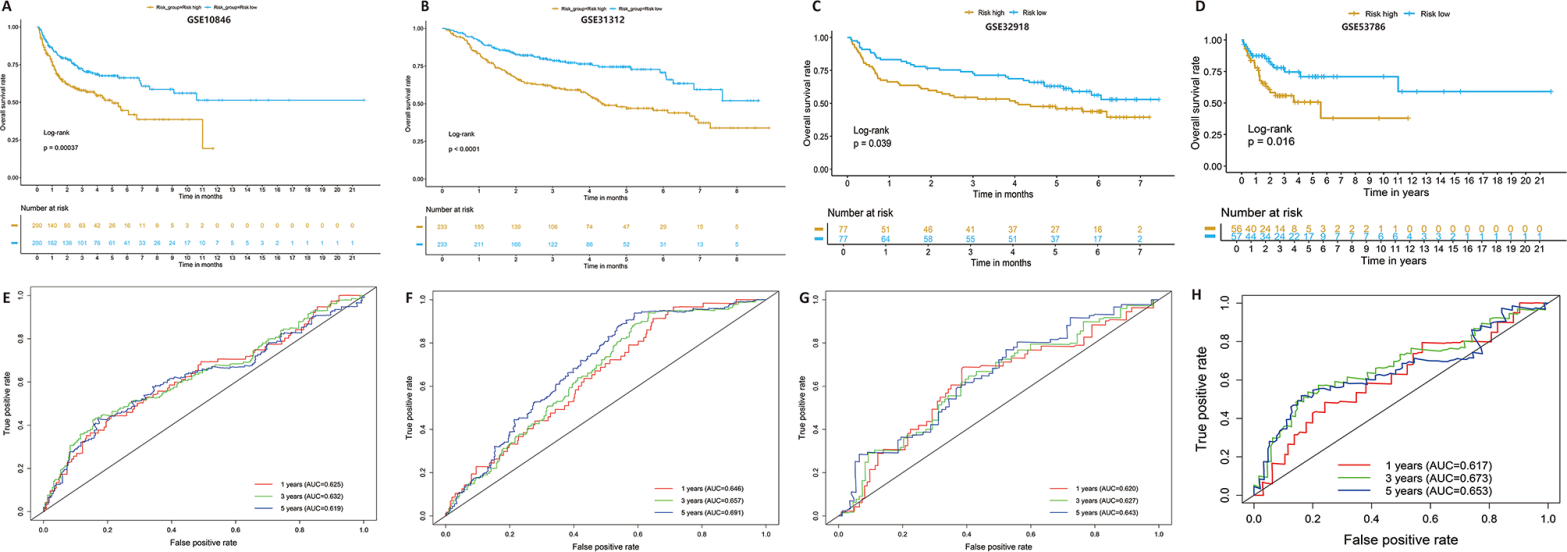
Validation of the LMRGs risk model. (**A**, **B**, **C**, **D**) Kaplan-Meier analyses of overall survival for patients in high and low risk groups in external validation cohorts, GSE10846, GSE31312, GSE32918 and GSE53786. (**E**, **F**, **G**,**H**) Time-dependent ROC analyses of the risk model in external validation cohorts

To further identify the prognostic related LMRGs, univariate Cox regression was first applied to the training cohort GSE181063 data set (*N* = 811) with the criteria of *P* < 0.05 and 238 prognostic related LMRGs were obtained. Then LASSO Cox regression model with 10-fold cross validation was further utilized to select the most powerful prognostic genes by minimizing the risk of over-fitting and 19 genes TMEM176B, LAYN, RAB6B, MMP9, ATAD3B, SLC2A11, CD3E, SLIT2, SLC2A13, SLC43A3, CD6, SIRPG, NEK6, LCP2, CTTN, CXCL2, SNX22, BCL6 and FABP4 were finally obtained (Fig. 2A, B). Patients were assigned to high-risk and low-risk groups according to the median risk score (Fig. 2 C). The prognosis of DLBCL patients in the low-risk group was better than that in the high-risk group in the train set (Fig. 2 C, middle). Survival curves indicated that DLBCL patients in the low-risk group had a significantly higher survival probability compared to the patients in high-risk group (*p* < 0.05) (Fig. 2D). ROC analysis showed that the area under the curve (AUC) at 1-,3-, 5-year was 0.741, 0.755 and 0.763 for the training set separately (Fig. 2E). To confirm the efficacy of the LMRGs survival model, we validated it in four external DLBCL cohorts including mRNA expression data from three microarray platform and one RNA-seq platform. Similarly, in three data sets from microarray platform, high-risk patients exhibited a significantly unfavorable prognosis, compared to low-risk patients (Fig. 3A, B, C; *p* < 0.001 for GSE10846 (*N* = 400) and GSE31312 (*N* = 466); *p* < 0.05 for GSE32918 (*N* = 154)). AUCs at 1-,3-, 5-year ranged from 0.619 to 0.691 (Fig. 3D, E, F). As for the data set from RNA-seq platform, the prognosis of patients in the low-risk group was better than that in the high-risk group although the difference between two groups was not significant in the TCGA-DLBCL cohort with less samples (Figure S1A; *p* = 0.084 for TCGA-DLBCL (*N* = 46)). AUCs at 1-,3-, 5-year was 0.674, 0.688 and 0.797 respectively (Figure S1B). Moreover, the risk score was positively associated with the International Prognostic Index (IPI) score and clinical stage (Figure S2). Clearly, our LMRGs survival model illustrated high predictive efficacy helpful in predicting the outcome of DLBCL patients.

### Impact of LRS on immune TME landscape

**Fig. 4:**
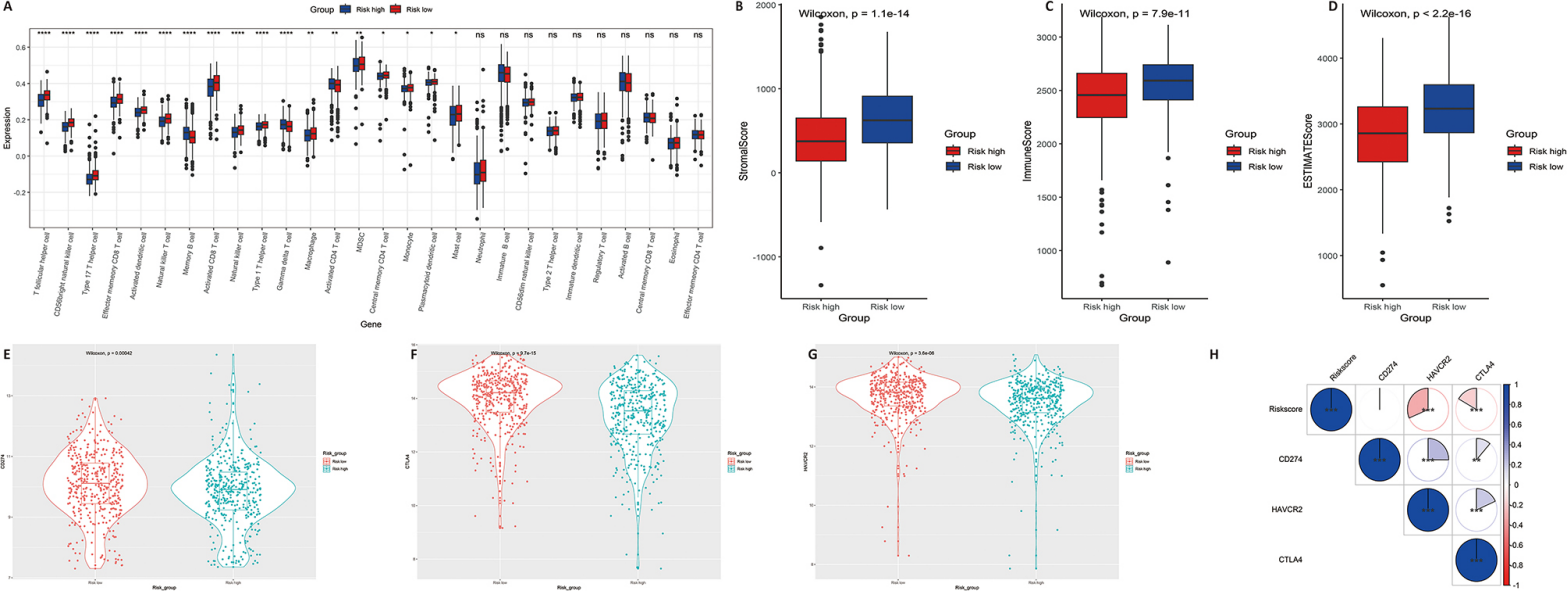
Tumor Microenvironment and immune cell infiltration analysis in train set GSE181063. (**A**) Differences between the high and low risk groups in the abundance of infiltrating immune cells. The red box: risk high; The blue box: risk low. (**B**,**C**,**D**) Differences in Stromal score, Immune Score and Estimate score between the high and low risk groups. (**E**,**F**,**G**) The expression of immune checkpoint molecules (PDL1,CTLA4,HAVCR2) were evaluated between high and low risk groups. (**H**) Correlation of risk score and immune checkpoint molecules (PDL1,CTLA4,HAVCR2)

Despite tumor cell per se, the microenvironment, including immune cells and stromal cells, is believed to play a critical role in determining the survival of DLBCL. Therefore, to decipher the possible mechanism that led to the distinct clinical outcome between high-risk and low-risk groups we evaluated the immune score, stromal score and ESTIMATE score between two groups. Significant differences in the infiltration of most immune cells between the two groups were the infiltration levels of activated CD8^+^ T cells, natural killer (NK) cells, natural killer T (NKT) cells and Macrophages were decreased in high-risk group (Fig. 4A). Accordingly, the stromal score (*P* < 0.001), immune score (*P* < 0.001) and ESTIMATE score (*P* < 0.001) were decreased in high-risk group (Fig. 4B–C). Furthermore, the expression of immune checkpoint molecules PDL1 (*P* < 0.001), cytotoxic T lymphocytes associated antigen-4 (CTLA-4) (*P* < 0.001), and T cell immunoglobulin and mucin domain containing protein-3 (TIM-3, HAVCR2) (*P* < 0.001) were also downregulated in high-risk patients (Fig. 4E–G) and the risk score was negatively associated with the expression of CTLA4 and HAVCR2 (Fig. 4H). In the external validation cohorts GSE181046, the immune score (*P* < 0.001) and ESTIMATE score (*P* < 0.001) were decreased in high-risk group (Figure S3 B, C). On the contrary, the expression of these immune checkpoint molecules were upregulated in DLBCL tissues compared with normal tissues (Figure S4). Taken together these data suggested that immunosuppression may not contribute to the poor survival outcome of patients in the high-risk group and other potential mechanisms should contributed to the decreased survival time of patients in the high-risk group.

### Functional enrichment analysis according to risk groups

**Fig. 5:**
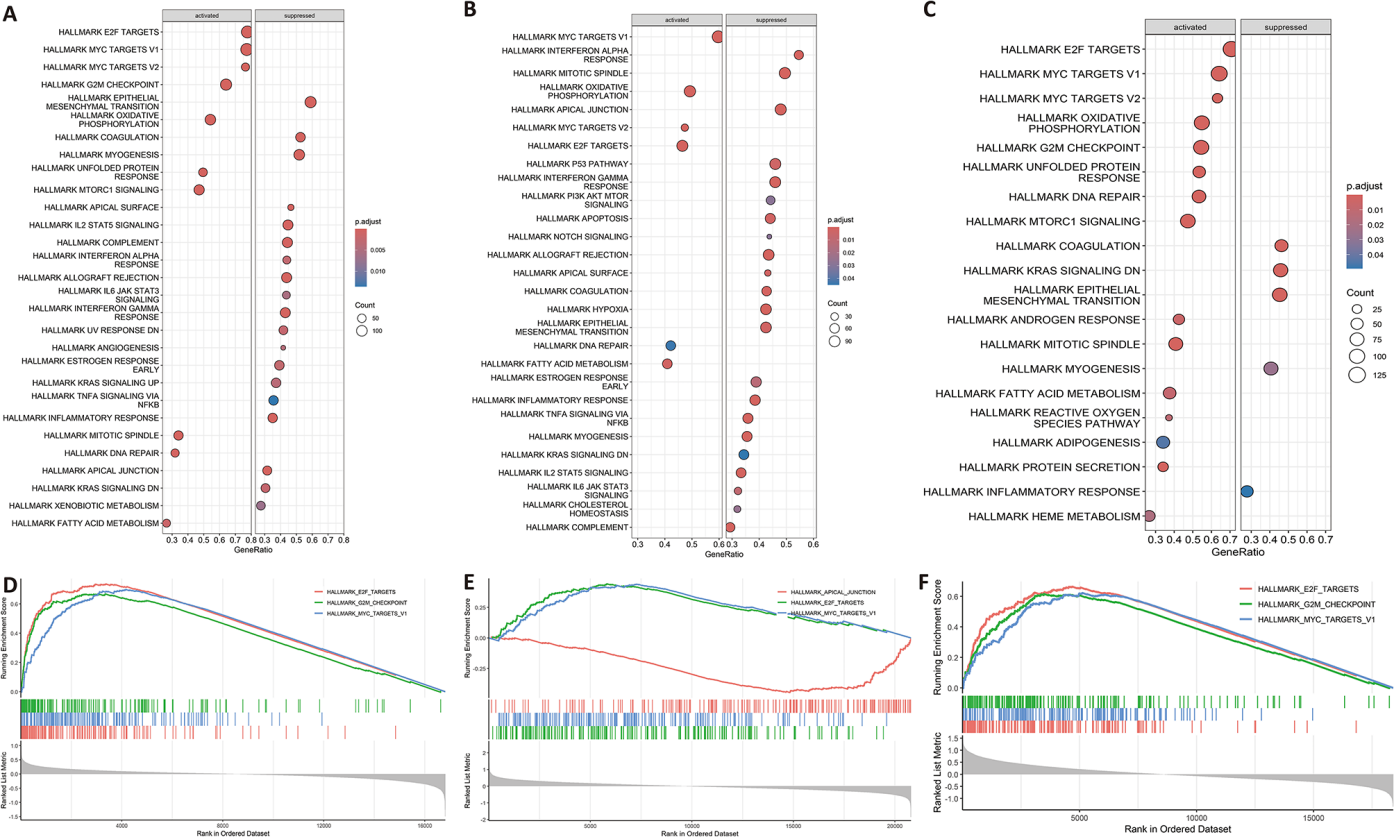
GSEA analysis of differential expressed genes between high and low risk group. (**A**,**D**) GSEA analysis of train set GSE181063 and Top3 activated pathway were HALLMARK E2F TARGETS, HALLMARK MYC TARGETS V1 and HALLMARK MYC TARGETS V2. (**B**,**E**) GSEA analysis of external validation set GSE10846 and Top3 activated pathway were HALLMARK MYC TARGETS V1, HALLMARK OXIDATIVE PHOSPHORYLATION and HALLMARK MYC TARGETS V2. (**C**,**F**) GSEA analysis of external validation set GSE32918 and Top3 activated pathway were HALLMARK E2F TARGETS, HALLMARK MYC TARGETS V1 and HALLMARK MYC TARGETS V2

We then performed GSEA enrichment analysisbetween the high-risk and low-risk groups in the training set and the validation set, respectively. In the training set, top activated pathway were Hallmark E2F targets, Hallmark MYC Targets V1 and Hallmark MYC Targets V2 (Fig. 5A, D). Consistently in the external validation set GSE10846 and GSE32918, top 3 activated pathway were Hallmark MYC Targets V1, Hallmark Oxidative Phosphorylation and Hallmark MYC Targets V2 (Fig. 5B, E) and Hallmark E2F Targets, Hallmark MYC Targets V1 and Hallmark MYC Targets V2 separately (Fig. 5C, F). This observation was also confirmed in TCGA-DLBCL cohort (Figure S4 C). While the GSEA result between normal and DLBCL patients sample showed the top activated pathways were Oxidative Phosphorylation, E2F targets, interferon-gamma response and epithelial mesenchymal transition (Figure S5) which was different from that of subtypes base on our risk model. Collectively, the activation of MYC targets genes is crucial for the poor survival outcome of patients in the high-risk group.

### Evaluating the therapeutic response in the high-risk and low-risk group

**Fig. 6:**
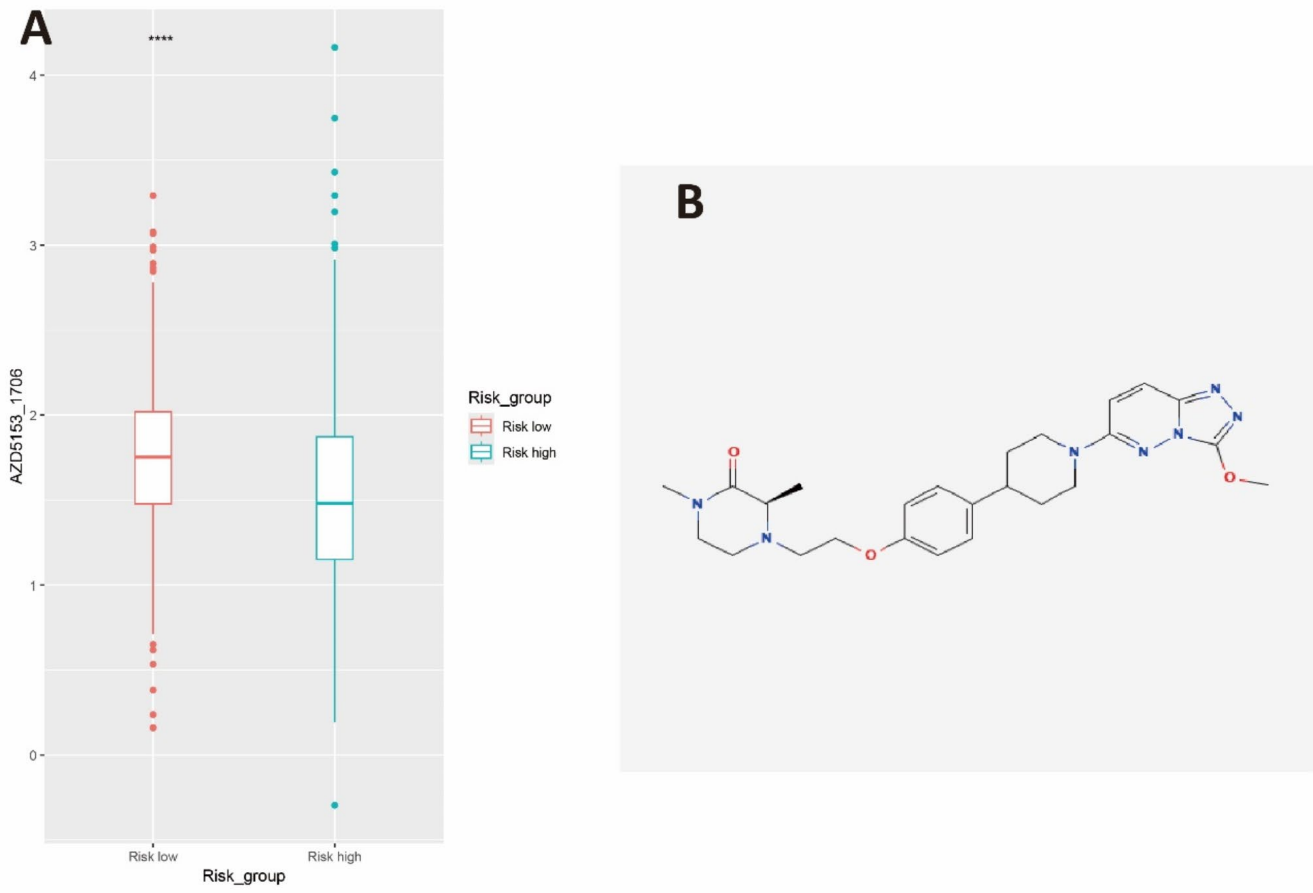
The screened drugs for DLBCL treatment in high and low risk groups. (**A**) IC 50 value of AZD5153 between high and low risk groups. (**B**) chemical structure of AZD5153

To further find possible drugs for patients in high-risk group based on our our LMRGs survival model, we estimate the chemotherapeutic response based on the half-maximal inhibitory concentration (IC50) available in the genomics of drug sensitivity in cancer (GDSC) database using the oncopredict algorithm. A total of 110 small molecular compounds with significantly different responses (*P* < 0.01) were identified between high-and low-risk groups in our study. Based on our GSEA analysis which indicated that the activation of MYC targets genes is crucial for the poor survival outcome of patients in the high-risk group, we choose AZD5153 which could inhibit the transcription of MYC and E2F by targeting BRD4 bromodomains (Rhyasen et al. 2016) for in vitro drug sensitivity analysis since the patients with high-risk based on our LMRGs survival model are more sensitive to AZD5153 as manifested by lower IC50 (Fig. 6A).

### In vitro evaluation of drug sensitivity

**Fig. 7:**
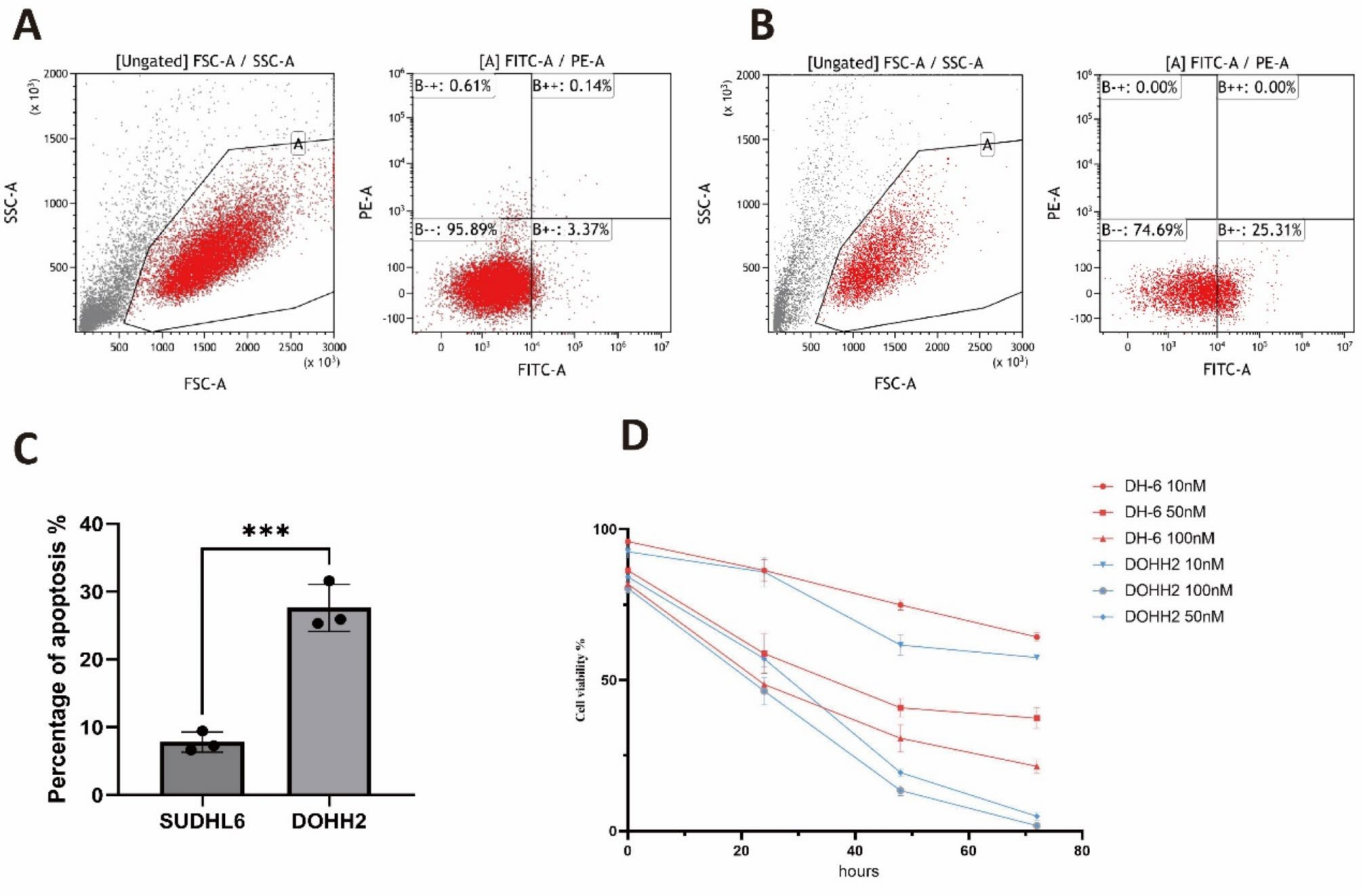
Cell viability and apoptosis analysis upon treatment of AZD5153. (**A**-**C**) DOHH2 (risk high) cells were sensitive to AZD5153 compared with SU-DHL-6 (risk low) cells as shown by more apoptotic cells treated under the same drug concentration. (**D**) Under the same drug concentration, SU-DHL-6 cells demonstrated high cell viability compared with DOHH2 cells

To validate the drug sensitivity of AZD5153, we divided the seventeen DLBCL cell lines into high and low risk groups based on the LMRGs survival model. DOHH2 (High risk) and SU-DHL-6 (Low risk) cells were seeded into 96-well plates and subjected to AZD5153 treatment. DOHH2 cells were more sensitive to AZD5153 treatment as the presence of more apoptotic cells (Fig. 7A-C) and compromised cell viability (Fig. 7D). Taken together these in vitro data suggested that the antitumor activity of AZD5153 might be specific to patients with certain lipid metabolism profiles.

## Discussion

Clinically, the prognosis of DLBCL patients is often prospectively predicted using the IPI score (Jelicic et al. 2023), however, due to the internal heterogeneous even patients with DLBCL at the same IPI risk stages demonstrate different clinical outcomes as manifested by approximately 30–40% relapse occurred within the first 2 years upon diagnosis with the R-CHOP regimen (Coiffier et al. 2010; Tavakkoli and Barta 2023) and even multiagent immunochemotherapy also fails to elicit a durable response in approximately one-third of patients with DLBCL (Schmitt et al. 2023). Since the traditional IPI score cannot adequately predict the prognosis of DLBCL and the molecular heterogeneity of DLBCL poses great challenges to predict disease progression and precision therapy, thus developing more reliable strategies for subtype identification and prognostic classification is in urgent need (Ruppert et al. 2020; Wight et al. 2018).

In the present study we established a 19 LMRGs risk model with high predictive efficacy. We initially obtained a total of 7286 LMRGs by integrating 176 individual collections from the MsigDB while studies on lipid metabolism signature in lung cancer, Glioma and Acute myeloid leukemia (AML) only include 1133,471 and 1045 genes respectively (Li et al. 2022, 2023; Zhu et al. 2022). This model was validated in four external DLBCL cohorts including mRNA expression data from three microarray platform and one RNA-seq platform and demonstrated a high predictive efficacy. Furthermore, based on the 19 LMRGs risk model we could determine novel subtypes with distinct lipid metabolism profiles and stratified patients into high and low risk status. Patients with high-risk score showed high IPI score and advanced clinical stage, suggesting a tight association with disease progression.

To unveil the possible mechanism that led to the distinct clinical outcome between high-risk and low-risk groups, we firstly analyzed the functions of the identified lipid metabolism genes separately and identified 3 hub genes (CD6, CD3E and LCP2) using STRING and Cytoscape. These hub genes are downregulated in the high-risk group while they are mainly responsible for the TCR mediated T cell activation which is distinct from immune check point (PDL1, CTLA4, HAVCR2) mediated immune repression (Deng et al. 2024; Stelzer et al. 2016; Edwards et al. 2023). Furthermore FABP4, a lipid chaperone protein, could promote ovarian cancer metastasis and drug resistance (Mukherjee et al. 2020). In response to lipid related inflammation, CXCL2 could be upregulated and promote cancer progression (Plastira et al. 2020; Zhang et al. 2023). On the other hand, some genes’ function such as SNX22 and SLC2A13 were scarcely reported in cancer. Since the hub genes are all related to the T cell inhibition we then evaluated the tumor immune microenvironment (TIM) by examining the types of infiltrated immune cells and calculating immune score, stromal score and ESTIMATE score since TIM is believed to play a critical role in progression of DLBCL (Sehn and Salles 2021; Colombo et al. 2022; Autio et al. 2021). Interestingly, in the high-risk group the infiltration levels of activated CD8^+^ T cells and effector memory CD8^+^ T cells were inhibited and the stromal score, immune score and ESTIMATE score were decreased. These results suggested insufficient immune cell infiltration. Recent studies highlight the significant roles of lipid metabolism reprogramming in regulating CD8^+^ T cell behaviors in tumor progression (Lim et al. 2022; Wang et al. 2023). Activated CD8^+^ T cell plays a critical role in anti-tumor immunity (St Paul and Ohashi 2020; Koh et al. 2023) and memory CD8^+^ T cell can maintain its proliferation property and make supplementation for effector CD8^+^ T cell (Sallusto et al. 2004). Except for insufficient CD8^+^ T infiltration, T cell exhaustion featured by upregulating inhibitory receptors like PD-1, CTLA-4 and TIM-3 also paved the way for the immune escape of tumor cell (Wherry 2011). However, the expression of these inhibitory molecules were downregulated in high-risk patients and the risk score was negatively associated with the expression of CTLA4 and TIM-3. On the contrary, the expression of these immune checkpoint molecules were upregulated in DLBCL tissues compared with normal tissues suggesting a very distinct immune landscape for patients with different subtypes of DLBCL. Taken together these data suggested that monoclonal antibody targeting immune checkpoint molecules may not be beneficial to the patients with certain lipid metabolism profiles and clinical application of immune checkpoint inhibitors (ICIs) should consider the specific gene expression background of the patients for precise medicine.

To figure out the possible mechanisms of the decreased survival in high-risk group, GSEA enrichment analysis was performed both in the training set and the validation set, respectively since GSEA would not leave out genes that was not statistically different but do possess biological function (Subramanian et al. 2005). Very consistently, the activation of MYC target genes was observed in the training set and in the validation set regardless of the source of the expression data (Microarray or RNA-seq) but not in the GSEA result between normal and DLBCL patients’ sample, suggesting the distinct mechanism base on the molecular subtypes of DLBCL (Song et al. 2023). Accordingly, a recent study unveiled a novel metabolic function of MYC in regulation of fatty acid synthesis in prostate cancer, indicate that inhibition of fatty acid synthesis by targeting MYC ACLY/ACC1/FASN axis may be a viable strategy for prevention and/or therapy of prostate cancer(Singh et al. 2021).

To provide specific drugs for patients with certain lipid metabolism profiles, we then estimate the chemotherapeutic response based on the IC50 available in the genomics of drug sensitivity in cancer (GDSC) database using the oncopredict algorithm (Maeser et al. 2021). A total of 110 small molecular compounds with significantly different responses were identified. Based on our GSEA analysis which indicated that the activation of MYC targets genes is crucial for the poor survival outcome of patients in the high-risk group, we choose AZD5153 which could inhibit the transcription of MYC and E2F by targeting BRD4 bromodomains (Rhyasen et al. 2016) as potential candidate and consistently, a recent study showed that BRD4 inhibition sensitizes diffuse large B-cell lymphoma cells (Schmitt et al. 2023). To validate the drug sensitivity of AZD5153, we divided the seventeen DLBCL cell lines into high and low risk groups based on the LMRGs survival model and choose DOHH2 (high-risk) and SU-DHL-6 (low-risk) cells for cell viability and apoptosis assays. The results showed that AZD5153 demonstrated a specific anti-tumor effect on DOHH2 cells. In summary we not only provide a model with high predictive efficacy for predicting the survival of patients with DLBCL but also demonstrated that drugs that compromising MYC target genes rather than ICIs may be beneficial to DLBCL patients with specific lipid metabolism status. These results provide clues for the possible mechanisms of inefficient ICIs for partial patients with DLBCL and uncovered potential drugs for these patients.

There are still some limitations in our study: First, although we used four external microarray cohorts and one RNA-seq cohort, more extensive validation using larger and diverse patient populations would strengthen the conclusions. Second, the possible mechanisms by which the MYC targets genes are activated in the high-risk group remained unclear. Therefore, future research studies should recruit more patients to validate the model. For clinical application PDX-mice model should be used to verified the efficacy of AZD5153 in high-risk DLBCL patients after which clinical trials could be implemented to confirm the anti-tumor effects of AZD5153 for high-risk patients. Fortunately, a recent study provided the details of AZD5153 clinical application such as the doze, MDT, pharmacodynamics and toxicities which provide us with valuable guide for our own clinical trials in the near future(Hamilton et al. 2023).

## Electronic supplementary material

Below is the link to the electronic supplementary material.

Supplementary Material 1: Figure S1. Validation of the LMRG prognostic model and DESA analysis result in TCGA-DLBCL (RNA-seq) cohort (A) Kaplan-Meier analysis of overall survival in high and low risk groups. (B) Time-dependent ROC analysis of the lipid metabolism risk model. (C) GSEA analysis of external validation set TCGA-DLBCL and Top3 activated pathway were HALLMARK MYC TARGETS V2, HALLMARK MYC TARGETS V1 and HALLMARK E2F TARGETS

Supplementary Material 2: Figure S2. The risk score is associated with clinical parameters of DLBCL patients. (A) Patients in the high-risk group have high IPI score. (B) DLBCL patients at advanced clinical stages (stage III and IV) have high risk score compared with patients at early clinical stages (stage I and II). (C) Comparison of risk score among patients at different clinical stages

Supplementary Material 3: Figure S3. Tumor Microenvironment analysis in external validation set GSE10846

Supplementary Material 4: Figure S4. Expression of immune checkpoint molecules in normal and DLBCL cohort The expression of immune checkpoint molecules (PDL1, CTLA4, HAVCR2) were evaluated between normal and DLBCL patients

Supplementary Material 5: Figure S5. GSEA analysis of differential expressed genes between normal and DLBCL samples

Supplementary Material 6

Supplementary Material 7

Supplementary Material 8

Supplementary Material 9

Supplementary Material 10

Supplementary Material 11

Supplementary Material 12

## Data Availability

No datasets were generated or analysed during the current study.
