# Supplementary figures and images for: Lipid metabolism-related gene signature predicts prognosis and unveils novel anti-tumor drugs in specific type of diffuse large B cell lymphoma
Source: Mol Med. 2024 Nov 13;30:210. doi: 10.1186/s10020-024-00988-4 (PMC11559131; doi:10.1186/s10020-024-00988-4)

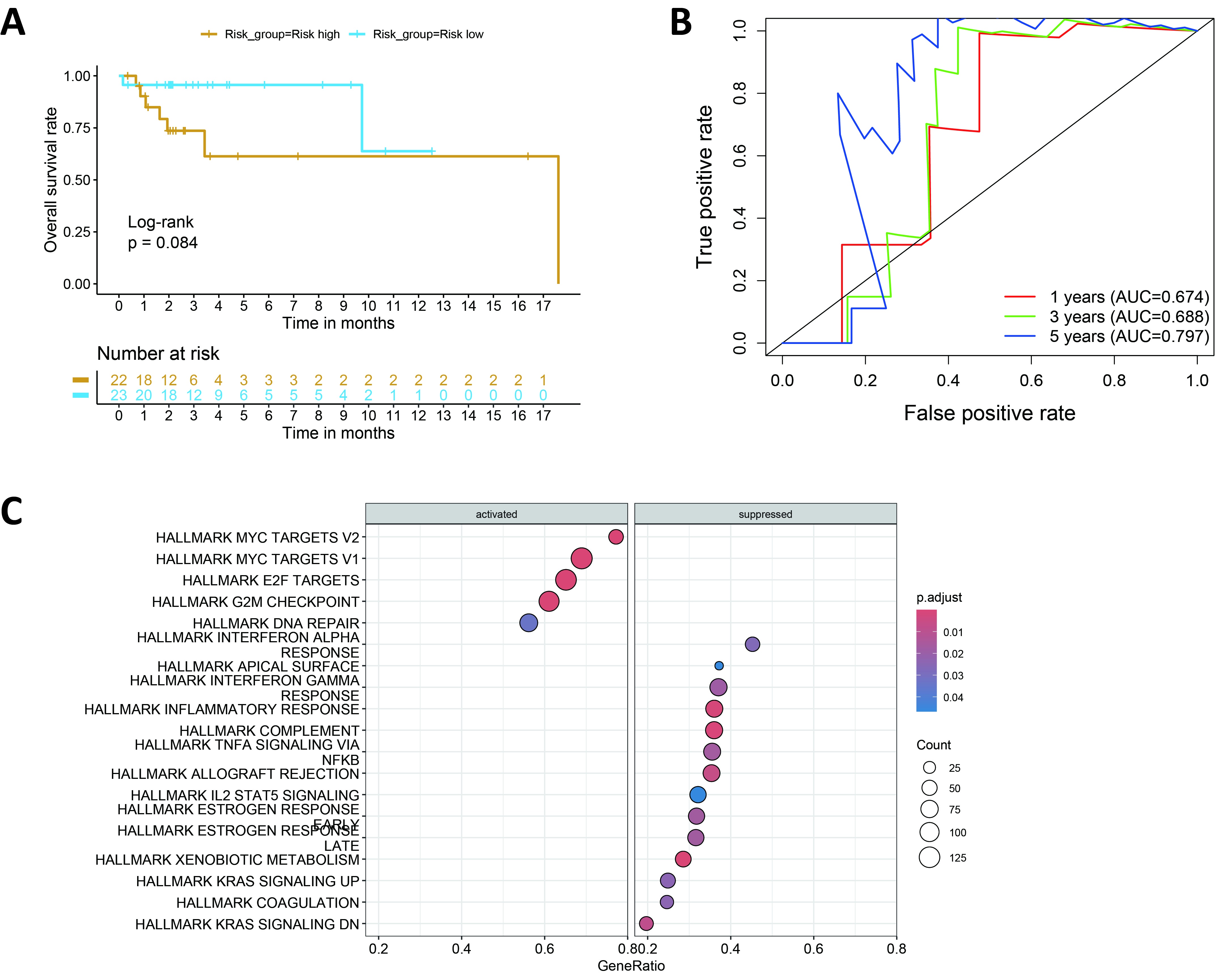

Supplement: Supplementary file 1 — Supplementary Material 1: Figure S1. Validation of the LMRG prognostic model and DESA analysis result in TCGA-DLBCL (RNA-seq) cohort (A) Kaplan-Meier analysis of overall survival in high and low risk groups. (B) Time-dependent ROC analysis of the lipid metabolism risk model. (C) GSEA analysis of external validation set TCGA-DLBCL and Top3 activated pathway were HALLMARK MYC TARGETS V2, HALLMARK MYC TARGETS V1 and HALLMARK E2F TARGETS [file 10020_2024_988_MOESM1_ESM.jpg]

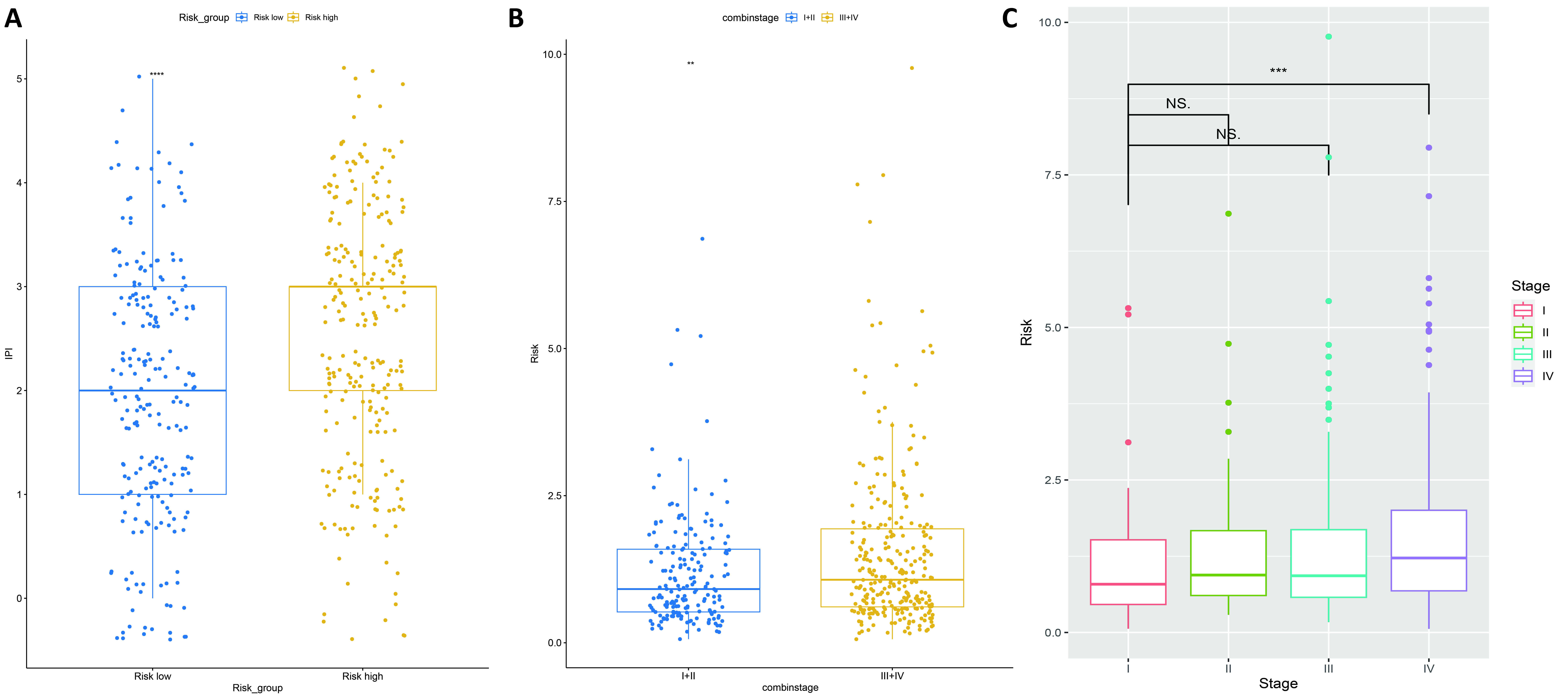

Supplement: Supplementary file 2 — Supplementary Material 2: Figure S2. The risk score is associated with clinical parameters of DLBCL patients. (A) Patients in the high-risk group have high IPI score. (B) DLBCL patients at advanced clinical stages (stage III and IV) have high risk score compared with patients at early clinical stages (stage I and II). (C) Comparison of risk score among patients at different clinical stages [file 10020_2024_988_MOESM2_ESM.jpg]

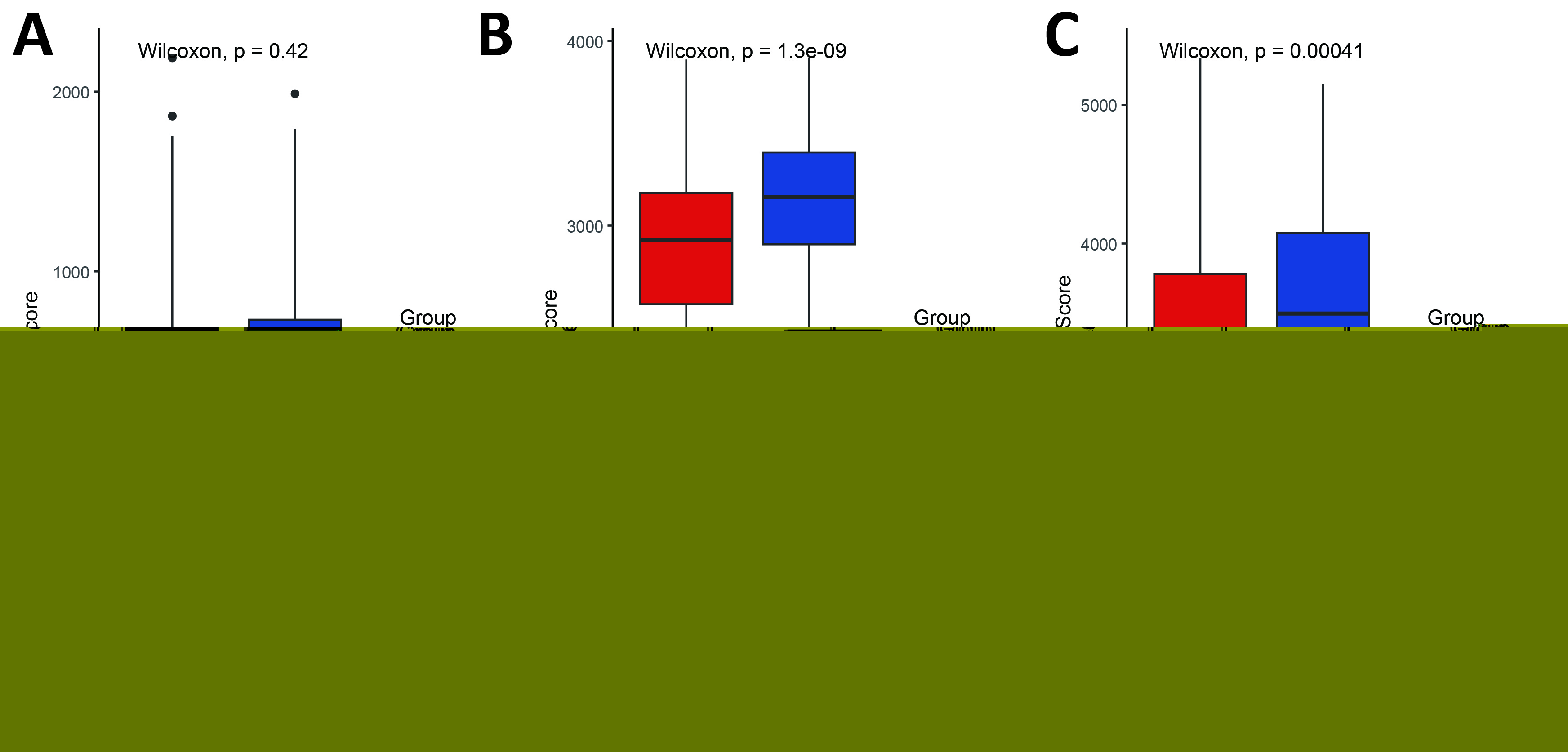

Supplement: Supplementary file 3 — Supplementary Material 3: Figure S3. Tumor Microenvironment analysis in external validation set GSE10846 [file 10020_2024_988_MOESM3_ESM.jpg]

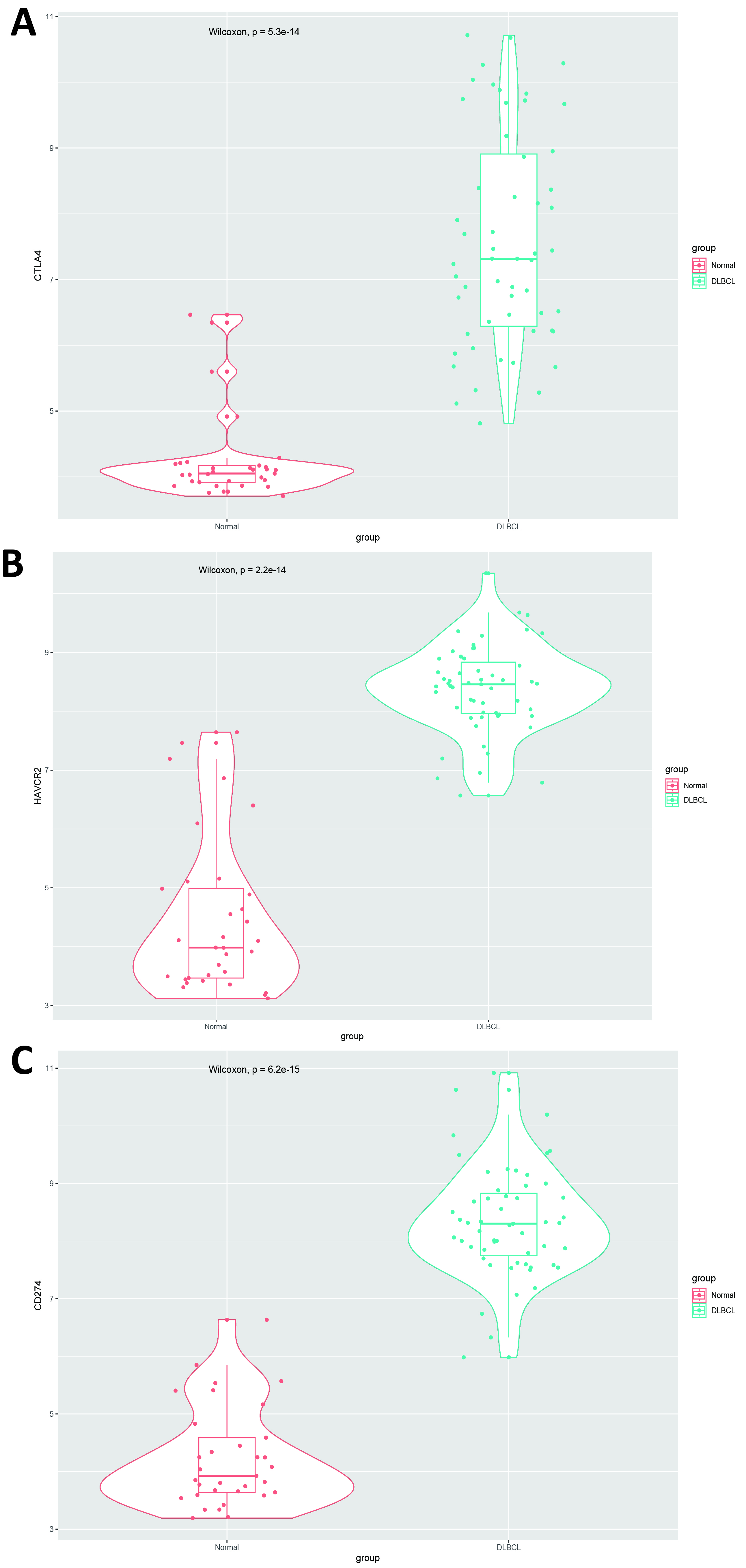

Supplement: Supplementary file 4 — Supplementary Material 4: Figure S4. Expression of immune checkpoint molecules in normal and DLBCL cohort The expression of immune checkpoint molecules (PDL1, CTLA4, HAVCR2) were evaluated between normal and DLBCL patients [file 10020_2024_988_MOESM4_ESM.jpg]

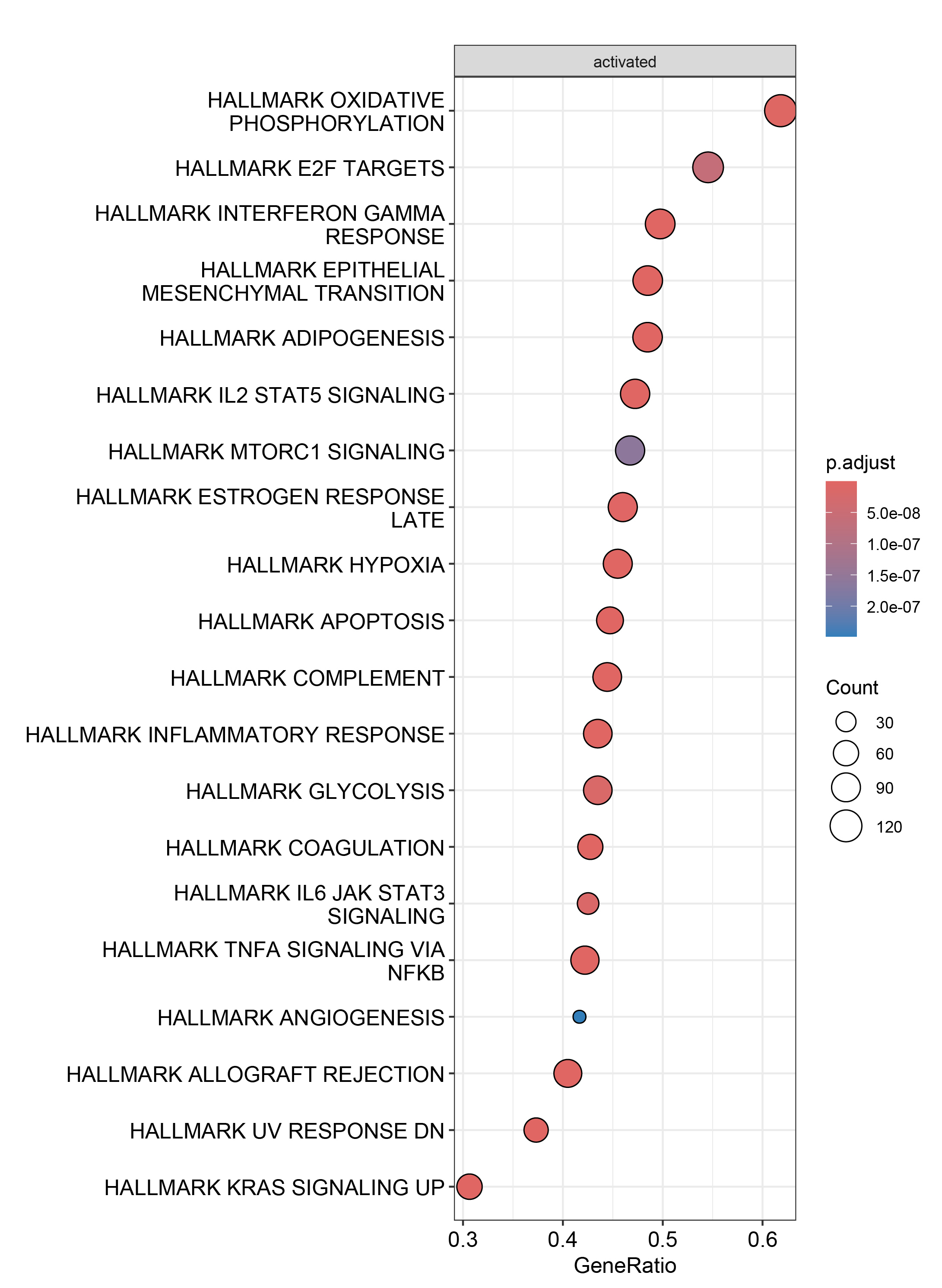

Supplement: Supplementary file 5 — Supplementary Material 5: Figure S5. GSEA analysis of differential expressed genes between normal and DLBCL samples [file 10020_2024_988_MOESM5_ESM.jpg]
